# Heterojunctions of Mercury Selenide Quantum Dots and Halide Perovskites with High Lattice Matching and Their Photodetection Properties

**DOI:** 10.3390/ma17081864

**Published:** 2024-04-18

**Authors:** Chengye Yu, Yufeng Shan, Jiaqi Zhu, Dingyue Sun, Xiaohong Zheng, Na Zhang, Jingshan Hou, Yongzheng Fang, Ning Dai, Yufeng Liu

**Affiliations:** 1 School of Materials Science and Engineering, Shanghai Institute of Technology, Shanghai 201418, China; yuchengye1999@163.com (C.Y.); sundy@sit.edu.cn (D.S.); zhengxiaohong@sit.edu.cn (X.Z.); nzhang@sit.edu.cn (N.Z.); houjingshan@hotmail.com (J.H.); 2 Hangzhou Institute for Advanced Study, University of Chinese Academy of Sciences, Hangzhou 310024, China; jiaqizhu@ucas.ac.cn (J.Z.); 216081175@mail.sit.edu.cn (N.D.); 3 State Key Labratory of Infrared Physics, Shanghai Institute of Technical Physics, Chinese Academy of Sciences, Shanghai 200083, China

**Keywords:** heterojunction semiconductor, HgSe, quantum dots, halide perovskite, ligand exchange

## Abstract

Heterojunction semiconductors have been extensively applied in various optoelectronic devices due to their unique carrier transport characteristics. However, it is still a challenge to construct heterojunctions based on colloidal quantum dots (CQDs) due to stress and lattice mismatch. Herein, HgSe/CsPbBr_x_I_3−x_ heterojunctions with type I band alignment are acquired that are derived from minor lattice mismatch (~1.5%) via tuning the ratio of Br and I in halide perovskite. Meanwhile, HgSe CQDs with oleylamine ligands can been exchanged with a halide perovskite precursor, acquiring a smooth and compact quantum dot film. The photoconductive detector based on HgSe/CsPbBr_x_I_3−x_ heterojunction presents a distinct photoelectric response under an incident light of 630 nm. The work provides a promising strategy to construct CQD-based heterojunctions, simultaneously achieving inorganic ligand exchange, which paves the way to obtain high-performance photodetectors based on CQD heterojunction films.

## 1. Introduction

Heterojunction semiconductors have been extensively applied in various active optoelectronic devices due to their unique characteristics of carrier regulation and redistribution [1]. Depending on the conditions, each heterostructuring step can thus proceed via a specific mechanism [2], such as epitaxial deposition [3], partial ion-exchange conversion [4], phase segregation [5], and induced fusion [6]. However, due to the limitations posed by the poor stability of nanomaterials with large specific surface areas and the lattice matching issues among heterogeneous materials [7,8]. The construction of heterojunctions with nanomaterials such as quantum dots, nanowires, and nanorods, which exhibit pronounced quantum effects, remains a challenge, thereby limiting their application in integrated optoelectronic devices like nanolasers, light-emitting diodes, solar cells, and photodetectors [9]. Especially for quantum dots with typical diameters ranging from 2 to 20 nm [10], it is more difficult to exchange the surface ligand to form a heterointerface on a small surface area. Therefore, it is urgent to exploit novel efficient methods for solving the problems of nanoscale semiconductor heterojunctions.

The early synthesis of chalcogenide group II–VI quantum dots was conducted in aqueous solutions under ambient temperatures and alkaline conditions. The resultant quantum dots demonstrated a broad distribution in particle size and exhibited lower quantum yields. Furthermore, a principal constraint of the aqueous synthesis approach was employing H_2_Te gas, which complicated the scalability of water-phase synthesis considerably. This was due to its complexity and time-consuming nature. It was not until 2003 that researchers such as Green [11] and subsequently Piepenbrock [12] employed metal salts and organic media for quantum dot synthesis, markedly enhancing the monodispersity and size uniformity of the quantum dots, while concurrently rendering the synthesis process more safe and straightforward. Nonetheless, CQDs synthesized via organic media are characterized by surfaces adorned with extensive long carbon chain ligands, including, but not limited to, oleic acid, oleylamine, and tributyl phosphate, which markedly impede the charge carrier transport within films, thereby precipitating a reduction in the efficiency of quantum dot optoelectronic devices [13]. Thus, it is essential to modify the surface of CQDs for improving the electrical properties. Ligand exchange is one of the most efficient surface modification strategy of CQDs, which can passivate surface defects to suppress carrier recombination [14]. However, the solid-state ligand exchange usually involves a complex multistep spin-coating process, followed by immersion in a solution consisting of organics with short carbon chains to acquire CQD films with a high mobility [15]. Substitution of the long carbon chain ligand with its short-chain counterpart typically induces surface cracking in the quantum dot film, thereby detrimentally impacting its photoelectric properties. Alternatively, solution-phase ligand exchange offers a straightforward and efficient modification strategy [16], in which CQDs dispersed in a non-polar solvent are mixed with polar solvents containing organics with short carbon chains to facilitate phase transfer and surface modification. 

However, the increment of mobility is limited owing to the poor conductivity of organics for quantum dots films. Therefore, inorganics are considered as an efficient ligand candidate [17]. Herein, HgSe CQDs with oleylamine ligands are synthesized via hot injection. The ligands on the quantum dots can been effectively exchanged to acquire smooth and compact CQD film using a halide perovskite precursor solution. Meanwhile, HgSe/CsPbBr_x_I_3−x_ heterojunctions with type I band alignment are acquired, derived from the minor lattice mismatching between CsPbBr_x_I_3−x_ and HgSe (~1.5%). The photoconductive detector based on HgSe/CsPbBr_x_I_3−x_ heterojunction presents a distinct photoelectric response under the incident light of 630 nm. Our work provides a promising strategy to construct QD-based heterojunctions, simultaneously achieving inorganic ligand exchange, which paves the way for obtaining high-performance photodetectors based on QD heterojunction films.

## 2. Materials and Methods

### 2.1. Materials

The starting materials of red mercuric iodide (HgI_2,_ Sigma-Aldrich, St. Louis, MO, USA, 99%), selenourea (CH_4_N_2_Se, Sigma-Aldrich, 98%), lead iodide (PbI_2_, Sigma-Aldrich, 98%), lead bromide (PbBr_2_, Sigma-Aldrich, 98%), cesium iodide (CsI, Sigma-Aldrich, 99%), oleylamine (OA, C_18_H_37_N, Sigma-Aldrich, 98%), N, N-dimethylformamide (DMF, Sigma-Aldrich, 99.8%, AR), and hexyl hydride (CH_3_(CH_2_)_4_CH_3_, Sigma-Aldrich, 98%, AR) were used. All of the chemicals were used as received, without further purification.

### 2.2. Synthesis of HgSe CQD

HgSe CQDs were synthesized utilizing the hot injection method. Initially, 1 mmol of HgI_2_ was completely dissolved in 10 mL of OA by heating the mixture to 100 °C for 1 h. Following the initial step, the temperature of the mixture was maintained at 115 °C for 30 min. Subsequently, 1 mmol of selenourea was dissolved in 10 mL of OA and heated to 140 °C under a nitrogen atmosphere for 22 h, resulting in the formation of a brown transparent liquid. A 5 mL aliquot of this solution was injected into the HgI_2_/OA solution, and the resultant mixture was reacted at 115 °C for 10 min, producing HgSe CQDs with an average diameter of approximately 4.5 nm. The vial containing the synthesized product was immediately removed from the glove box and cooled in ice water. Thereafter, methanol was added in a controlled quantity to precipitate the quantum dots, which were then isolated via centrifugation; residual particles were dissolved in a hexane solvent. All of the synthesized CQDs were meticulously stored in a nitrogen-purged glove box.

### 2.3. Solution-Phase Ligand Exchange

This procedure was executed within a glove box under solution-phase conditions. The ion exchange solution was meticulously prepared through the dissolution of PbI_2_ (0.133 mmol), CsI (0.007 mmol), and PbBr_2_ (0.013 mmol) in DMF. A colloidal quantum dot solution in octane (10 mg·mL^−1^) was combined with the exchange solution at a 1:1 volume ratio. The resultant mixture was vigorously agitated for 120 s, ensuring complete transfer of the CQDs to the DMF phase. Subsequently, 1 mL of toluene was introduced, followed by centrifugation to isolate the black precipitate from the bottom layer, which was then preserved for storage.

### 2.4. Device Fabrication

The electrode pattern was fabricated on a Si substrate coated with 280 nm of SiO_2_, employing standard photolithography and radio-frequency sputter deposition techniques. Layers of titanium (15 nm) and gold (45 nm) were sequentially deposited to form the electrodes. The electrode configuration featured four pairs of interdigitated, evaporated gold fingers, each characterized by a width of 20 μm, a gap of 100 μm, and a length of 250 μm. These electrodes spanned an effective area of 0.25 mm by 0.98 mm. Before the application of colloidal quantum dots, the substrate surface was treated with an oxygen plasma degumming machine for 30 s to ensure optimal adhesion. Subsequently, the colloidal quantum dot solution was applied to the substrate via either drop-casting or spin-coating techniques to achieve a film of approximately 150 nm in thickness.

### 2.5. Characterizations

Grazing-incidence X-ray diffraction data were meticulously acquired employing Rigaku Smartlab 9kw instruments (GIXRD, Smartlab 9KW, Rigaku, Tokyo, Japan) which operated at an optimized condition of 40 kV and 150 mA. The experimental parameters were precisely defined with a step size of 0.01, an angle range of 10–80°, a scanning speed of 2°/min, and a scanning angle meticulously set at 0.5°. The surface and cross-sectional morphology of HgSe films were comprehensively characterized via field emission scanning electron microscopy (SEM, Gemini 300, ZEISS, Oberkochen, Germany ). For a detailed analysis of HgSe CQDs pre- and post-exchange, transmission electron microscopy (TEM, JEM-2100F, JEOL, Tokyo, Japan) operating at an accelerated voltage of 200 kV, was meticulously employed. The infrared absorption spectrum was meticulously analyzed employing a Bruker VERTEX 80 spectrometer (80V, VERTEX 80V, Bruker, Saarbruecken, Germany). All of the experiments were systematically conducted under controlled ambient conditions, employing a consistent AFM probe, with the temperature meticulously maintained at 25 °C and relative humidity at approximately 25%.

## 3. Results and Discussion

**Figure 1:**
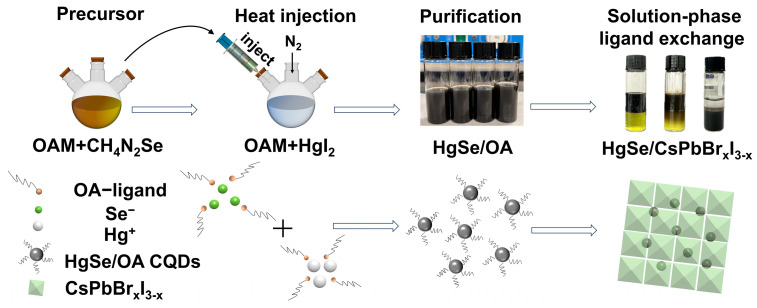
Schematic diagram of HgSe quantum dot synthesis and solution-phase ligand exchange.

The fabrication of HgSe CQDs and the solution-phase ligand exchange method are depicted in Figure 1. Initially, mercury and selenium were dissolved in OA, preparing the precursors. The selenium precursor was then cooled to ambient conditions, whereas the mercury precursor remained at 115 °C. The selenium was gradually introduced into the mercury solution, culminating in the synthesis. The resultant HgSe CQDs, purified via centrifugation and extraction, were then mixed in precise ratios with CsPbBr_x_I_3-x_ solutions. A vortexing step facilitated the HgSe CQDs’ transition to the DMF phase, after which they were collected by centrifugation and redispersed in a BTA-DMF solution.

**Figure 2:**
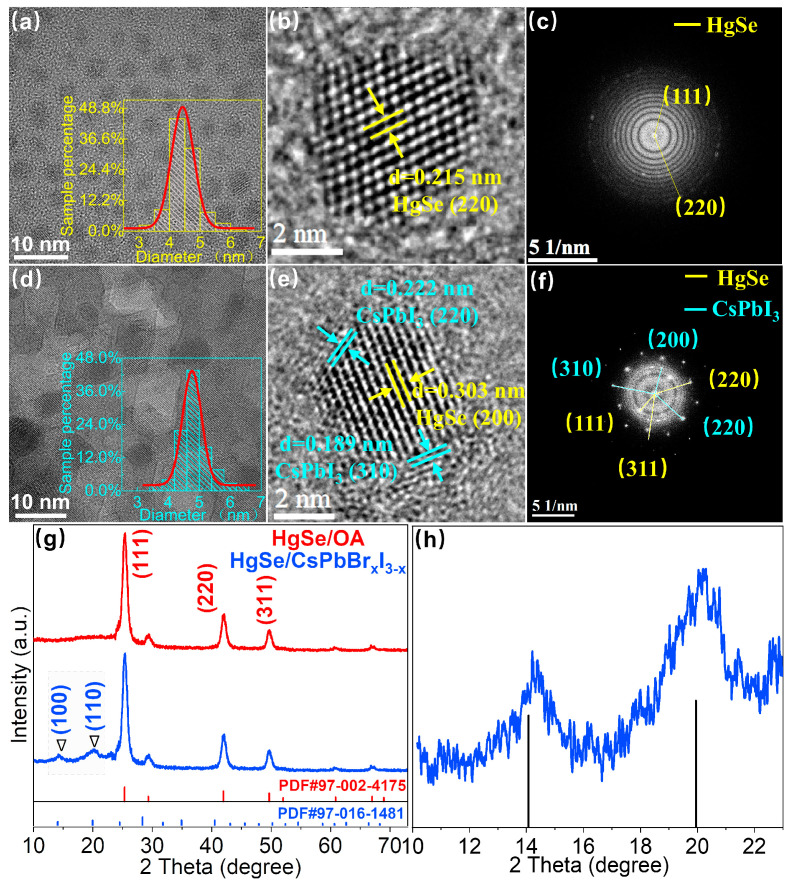
(**a**–**c**) SEM, particle size statistics and FFT images of HgSe/OA. (**d**–**f**) SEM, particle size statistics and FFT images of HgSe/CsPbBr_x_I_3−x_. (**g**) GIXRD patterns of HgSe/OA and HgSe/CsPbBr_x_I_3−x_ films. (**h**) HgSe/CsPbBr_x_I_3−x_ film with locally amplified GIXRD spectra.

To verify the formation of the HgSe/CsPbBr_x_I_3−x_ heterostructure, the exchanged materials were characterized by TEM. Figure 2a–c indicates that prior to the solution-phase ligand exchange, the HgSe/OA quantum dots exhibited a uniform size distribution and excellent monodispersity. Additionally, Fast Fourier Transform Algorithm (FFT) images revealed diffraction spots corresponding to crystal planes of (111), (220), and (311), as observed in the XRD data. The formation of HgSe/CsPbBr_x_I_3−x_ heterostructures following ligand exchange is depicted in Figure 2e. The FFT image vividly displays the distinct diffraction spots formed by the HgSe/CsPbBr_x_I_3−x_ heterostructures, highlighting their perfect formation (Figure 2f). This observation further suggests that the low lattice mismatch facilitates the epitaxial growth of the perovskite matrix around the quantum dots. Elemental mapping images further confirm the coexistence of Hg, Se, Cs, Pb, I, and Br elements within the heterostructures (Figure S1). Statistical analysis reveals that HgSe/OA CQDs have an average size of 4.42 ± 0.16 nm. Upon ligand exchange with a perovskite precursor, the quantum dot size marginally increased to 4.72 ± 0.05 nm. This slight size variation before and after ligand exchange corroborated the XRD and infrared absorption data, indicating no significant change in the size of HgSe CQDs.

As depicted in Figure 2g, the sphalerite crystal structure of HgSe CQDs remained intact before and after the ligand exchange, indicating that our employed liquid phase ligand exchange method did not disrupt the original crystal structure [18]. The grazing incidence X-ray diffraction (GIXRD) pattern of the HgSe/CsPbBr_x_I_3−x_ film showed an additional but weak CsPbBr_x_I_3−x_ signal compared with the OA-capped HgSe CQD film; the weakness may be due to the low concentration or non-ideal crystallization of CsPbBr_x_I_3−x_ [19]. Through amplification and comparative analysis, it is evident from Figure 2h that compared with the standard contrast card of pure phase CsPbI_3_ perovskite, the measured diffraction peak at 0.16° shifted towards a larger angle, suggesting partial replacement of I atoms by Br atoms with a smaller radius, resulting in a lattice expansion. A small quantity of bromine atoms instead of iodine atoms was also favorable for the stable existence of our perovskite matrix in the natural environment. By applying Bragg’s law (nλ=2dSinθ and $a=d\times \sqrt{h^{2}+k^{2}+l^{2}}$) [20], it was calculated that the lattice constant of the perovskite within the heterostructure that formed after ligand exchange was 6.182 Å (Table S1), whereas the lattice constant of the synthesized HgSe quantum dots measured 6.086 Å. The resulting lattice mismatch between these two materials amounted to approximately 1.5%, thereby demonstrating the potential for the epitaxial growth of CsPbBr_x_I_3−x_ around HgSe CQDs.

**Figure 3:**
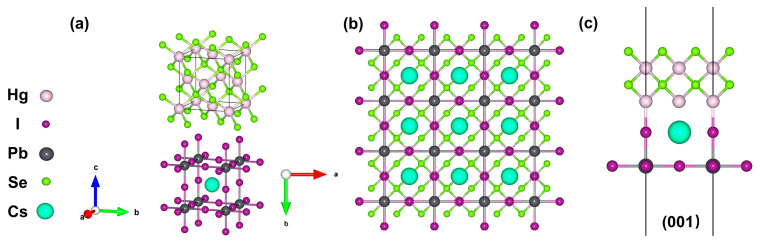
(**a**) The crystal structures of HgSe and CsPbI_3_. (**b**) Top view of the HgSe/CsPbI_3_ structure (001) surface. (**c**) Side view of the HgSe/CsPbI_3_ structure (001) surface.

The molecular structural schematic of the HgSe/CsPbI_3_ heterostructure is presented in Figure 3. As shown in Figure 3a, both HgSe and CsPbI_3_ crystallized in a cubic system. According to previous reports, if a small number of Br atoms replace the I atoms, the stability of CsPbI_3_ can be increased, and the lattice constant of CsPbI_3_ will be reduced [21]. Through the analysis of the relative peak shifts in the GIXRD diffraction, the lattice constant of the synthesized perovskite structure was determined to be 6.182 Å, a value closely approaching that of the lattice constant of HgSe quantum dots (6.086 Å). The exceedingly low lattice mismatch between HgSe and CsPbBr_x_I_3−x_ (~1.5%) suggested a pronounced epitaxial trend between the two materials. In this study, the (001) epitaxial interface was identified as the most probable based on reasonable considerations. The stable (001) epitaxial interface consisted of the HgSe (001) surface and the CsPbI_3_ (001) surface terminated by “PbI_2_” (Figure 3b,c). Therefore, the presence of the HgSe/CsPbBr_x_I_3−x_ heterostructure was a ration. 

**Figure 4:**
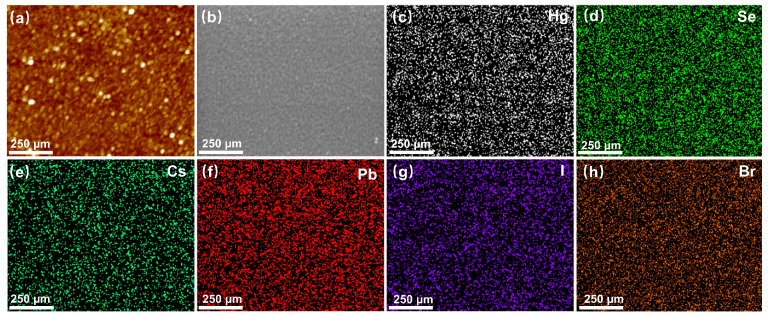
(**a**) AFM and (**b**) FESEM images of the HgSe/CsPbBr_x_I_3−x_ quantum dots film. (**c**–**h**) Elemental mapping of the HgSe/CsPbBr_x_I_3−x_ quantum dot film (scale bar, 250 μm).

The AFM and FESEM images in Figure 4a,b demonstrate the compact surface, uniform grain size, and relatively flat nature of the HgSe/CsPbBr_x_I_3−x_ quantum dot film after ligand exchange. The EDS element mapping in Figure 4c–h reveals that following ligand exchange, the HgSe/CsPbBr_x_I_3−x_ quantum dot film exhibited an even distribution of Hg, Se, Cs, Pb, I, and Br elements throughout the film.

**Figure 5:**
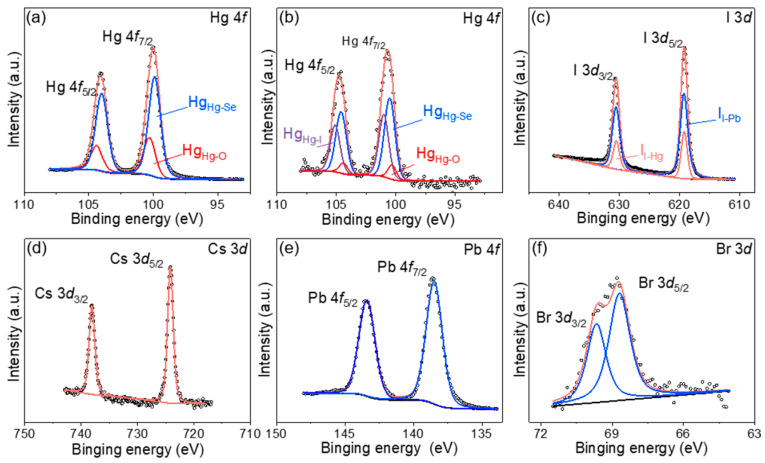
(**a**) The XPS spectra of Hg-4f orbitals of the HgSe/OA film. (**b**–**f**) XPS spectra of Hg-4f, I 3d, Cs 3d, Pb 4f, and Br 3d of HgSe/CsPbBr_x_I_3−x_ films.

To ascertain the chemical states of the constitutive elements in the quantum dot thin films before and after ligand exchange, X-ray photoelectron spectroscopy (XPS) measurements were conducted. The XPS elemental analysis confirmed the presence of Hg and Se in the CQDs films prior to ligand exchange, and additionally revealed Cs, Pb, I, and Br in the CQDs films following the exchange (Figure S2). The XPS spectral data have been calibrated using the standard C1s peak at 284.8 eV. The peak fitting results for the area ratios of d_3/2_ to d_5/2_ and f_5/2_ to f_7/2_ were approximately 2:3 and 3:4, respectively, consistent with the fundamental principles of orbital splitting. As depicted in Figure 5a,b, signals from the Hg 4f orbitals pre- and post-ligand exchange were split while peaks from the Hg-O bonds were obtained at 100.3 eV and 104.4 eV respectively [22,23]. The Hg-Se bond exhibited peaks at 99.9 eV and 104 eV prior to exchange, while the Hg-I bonds displayed peaks at 101 eV and 105.1 eV [24], which were sufficiently distinct from the Hg 4f track signals after exchange. Furthermore, the Hg-Se bond peaks shifted towards higher binding energy values of 100.5 eV and 104.6 eV following exchange, indicating an influence from the formation of the perovskite matrix on these bonds. Figure 5c illustrates the post-ligand exchange delineation of the I 3d orbital signal, revealing peak positions at 619.2 eV and 630.5 eV corresponding to the I-Pb and I-Hg bonds, respectively [25,26]. This finding is in concordance with the Hg-I bond separation, as inferred from the Hg 4f orbital signal, thus affirming the structural integrity of the bonds. Figure 5d–f illustrate orbital signals for Cs-3d, Pb-4f, and Br-3d detected in the quantum dots film after ligand exchange [27,28,29,30], conclusively evidencing the formation of perovskite within the heterostructure.

**Figure 6:**
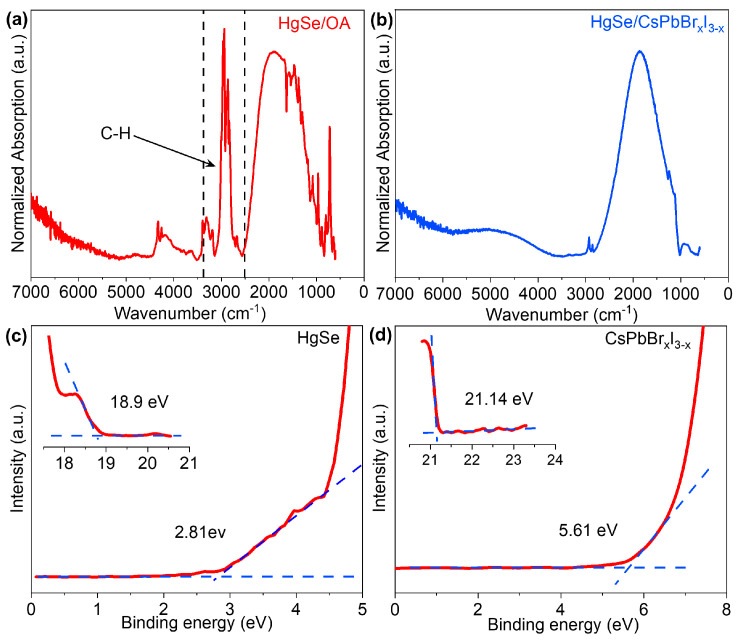
(**a**) Absorption spectra of HgSe/OA CQDs. (**b**) Absorption spectra of HgSe/CsPbBr_x_I_3−x._ (**c**) Valence band spectrum, and work function of HgSe quantum dots film. (**d**) Valence band spectrum and work function of CsPbBr_x_I_3−x_.

Figure 6a,b depicts the infrared absorption spectra of HgSe/OA and HgSe/CsPbBr_x_I_3−x_, respectively. The presence of an intraband absorption peak in the range of 1000~2500 cm^−1^ for HgSe/OA quantum dots signified stable doping, while interband absorption occurred at higher energies [31,32]. The characteristic organic peak associated with OA ligands was observed around 3000 cm^−1^ [33]. After the ligand exchange in the liquid phase, there was minimal alteration observed in the absorption peak of HgSe/CsPbBr_x_I_3−x_. However, a significant reduction was observed in the characteristic organic peak of OA ligands around 3000 cm^−1^. The observation underscored that subsequent to the establishment of the HgSe/CsPbBr_x_I_3−x_ heterostructures, the OA ligands present on the surface of the CQDs were entirely supplanted.

The work function of HgSe quantum dots and CsPbBr_x_I_3−x_ and the energy difference between the Fermi level and valence band maximum (VBM) can be obtained from the UV photoelectron spectroscopy (UPS) results, respectively. By integrating the band gap and UPS findings (Figure 6c) derived from the absorption spectrum (Figure 6a), the conduction band minimum (CBM) and valence band maximum (VBM) of HgSe CQDs could be determined. Based on the previous determination of the band gap for the CsPbBr_x_I_3−x_ [34,35] and UPS (Figure 6d) test results, the positions of the CBM and VBM within the perovskite were successfully identified. For the purpose of facilitating precise identification, the vacuum level was set to 0 eV in this instance. The relevant computational results are presented in Table 1**.**

**Figure 7:**
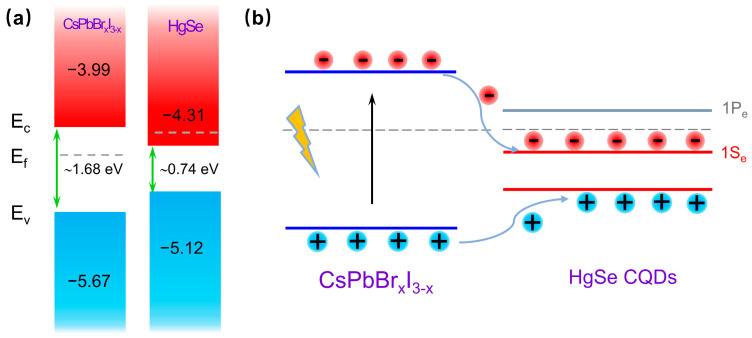
(**a**). Diagram of type-I band arrangement of HgSe/CsPbBr_x_I_3−x_. (**b**). Perovskite produces photogenerated charge carriers that transfer from CsPbBr_x_I_3−x_ to HgSe CQDs schematic.

Through a comprehensive analysis of the infrared absorption and UPS data, the precise energy band alignment of the HgSe/CsPbBr_x_I_3−x_ heterostructures was meticulously defined. Owing to the fact that the bandgap of HgSe CQDs was significantly narrower than that of CsPbBr_x_I_3−x_, forming a type-I band structure that was expected to be constrained by the holes and electrons of the larger band gap CsPbBr_x_I_3−x_ [36,37] (Figure 7a). When the perovskite was excited by photon energy exceeding its band gap, photogenerated carriers were generated in the perovskite and transferred from the CsPbBr_x_I_3−x_ to HgSe CQDs (Figure 7b).

**Figure 8:**
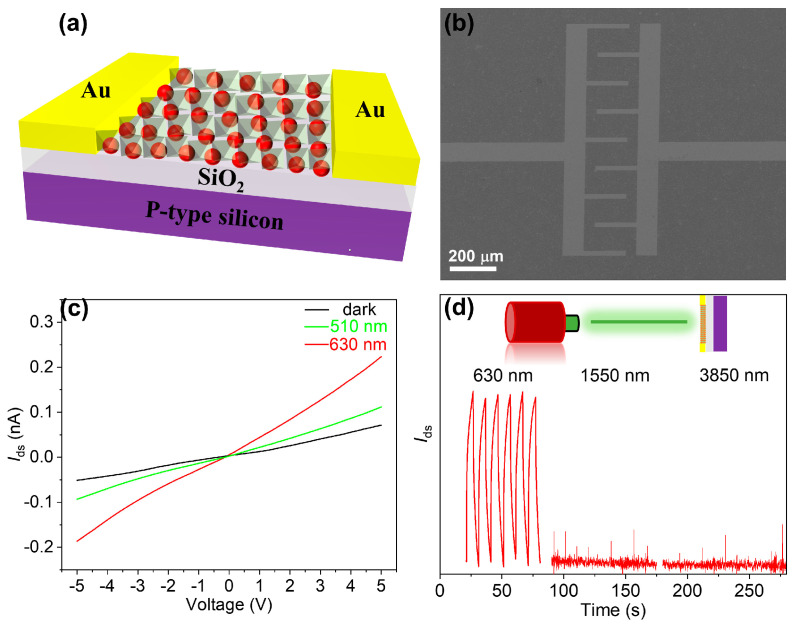
(**a**) Schematic diagram of HgSe/CsPbBr_x_I_3−x_ photoconductive device. (**b**) SEM image of interdigital electrode in photoconductive detector (Scale bar, 200 μm). (**c**) I–V characteristics of HgSe/CsPbBr_x_I_3−x_ devices under dark field, 510 nm and 630 nm LED light sources. (**d**) I–T characteristics of HgSe/CsPbBr_x_I_3−x_ devices under 630 nm, 1550 nm, and 3850 nm laser light sources.

In order to investigate the photogenerated carrier transport properties in materials exhibiting type-I structures, a photoconductive device was fabricated. Schematic illustrations of the device structure are presented in Figure 8a,b. The device demonstrated an exceptional response to the visible light spectrum (Figure 8c), primarily attributed to its utilization of perovskite materials. Through a comparative analysis of the device’s on–off characteristics, it was evident that perovskite showed a remarkably swift response owing to its elevated carrier mobility, thereby resulting in a square waveform switching curve (Figure S3). Conversely, for devices based on HgSe/CsPbBr_x_I_3−x_ type-I heterostructures, illumination with a visible light spectrum induced the transfer of carriers generated within the perovskite matrix into HgSe quantum dots. Consequently, the carrier lifetime experienced a significant enhancement within the visible spectral range of the device. This phenomenon was evident in the switch curve as it exhibited a deceleration in response time and deviated from its typical square wave. At an ambient temperature, HgSe CQDs exhibited an exceptional detection performance within the 1550 nm wavelength range and could also achieve efficient mid-wave infrared detection at low temperatures [38,39]. Conversely, the HgSe/CsPbBr_x_I_3−x_ devices exhibited a complete loss of infrared detection functionality (Figure 8d), a phenomenon that underscored the pronounced effect of carrier confinement within the type-I heterostructures engendered by photogenerated carriers [40].

## 4. Conclusions

In summary, HgSe CQDs with monodispersion and a uniform size were synthesized using the hot injection method. The TEM images showed that the synthesized HgSe CQDs had an average diameter of 4.42 ± 0.16 nm. The HgSe/CsPbBr_x_I_3−x_ heterostructure was fabricated using the solution-phase ligand exchange method. Based on the UPS and infrared absorption spectra analysis, the band arrangement was determined to be type I. Furthermore, a photoconductive device was fabricated to investigate the photoelectric properties of HgSe CQDs at their characteristic wavelengths, confirming the effective recombination of photogenerated carriers. 

## Figures and Tables

**Table 1:** Band gap, VBM, and CBM of HgSe and CsPbBr_x_I_3−x_ calculated by the UPS and absorption spectrum.

Samples	Band Gap (eV)	Work Function (eV)	VBM (eV)	CBM (eV)
HgSe CQDs	0.74	−2.3	−5.12	−4.31
CsPbBr_x_I_3−x_	1.68	−0.06	−5.67	−3.99

## Data Availability

Data are contained within the article and Supplementary Materials.

## Supplementary Materials

The following supporting information can be downloaded at: https://www.mdpi.com/article/10.3390/ma17081864/s1. Figure S1: EDS images of elements Hg, Se, Cs, Pb, I, and Br in the HgSe/CsPbBr_x_I_3−x_ heterostructure. Table S1: Calculation of lattice constants for CsPbBr_x_I_3−x_ crystals. Figure S2: Total XPS spectra of HgSe and HgSe/CsPbBr_x_I_3−x_. Figure S3: I–T switching curves of CsPbBr_x_I_3−x_.
