# Supplementary material for: Heterojunctions of Mercury Selenide Quantum Dots and Halide Perovskites with High Lattice Matching and Their Photodetection Properties
Source: Materials (Basel). 2024 Apr 18;17(8):1864. doi: 10.3390/ma17081864 (PMC11051518; doi:10.3390/ma17081864)
Supplement: Supplementary file 1 [file materials-17-01864-s001.zip › materials-2937581-supplementary.pdf]

Supporting Information

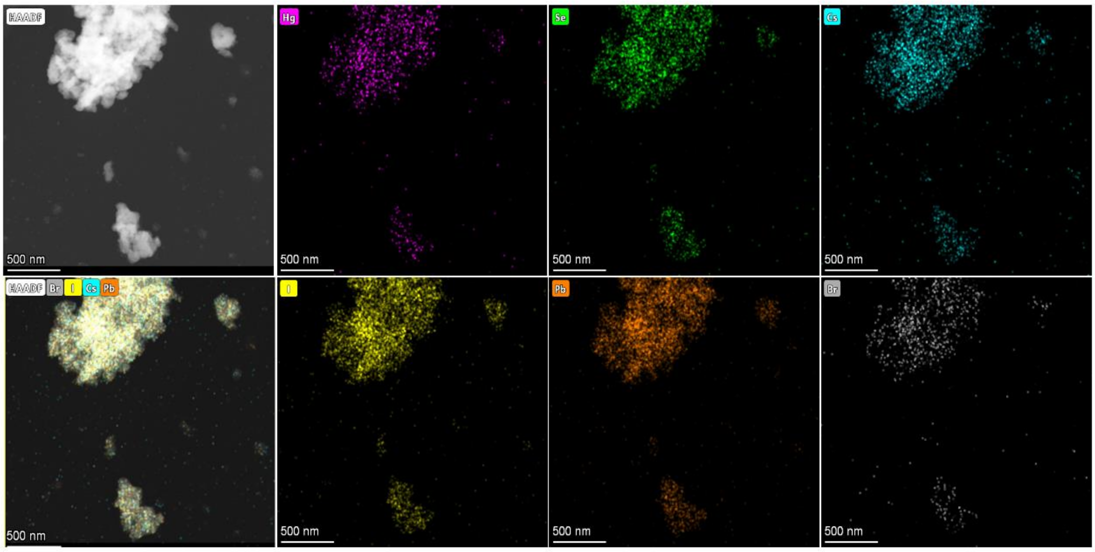

Figure S1. EDS images of elements Hg, Se, Cs, Pb, I, and Br in the HgSe/$CsPbBr_xI_{3-x}$ heterostructure

Substance	ϴ	Sinϴ	$d_{(110)}$（Å）	a（Å）
$CsPbI_3$	9.9745	0.173	4.4473	6.289
$CsPbBr_xI_{3-x}$	10.135	0.176	4.3715	6.182

Table S1. Calculation of lattice constants for $CsPbBr_xI_{3-x}$ crystals

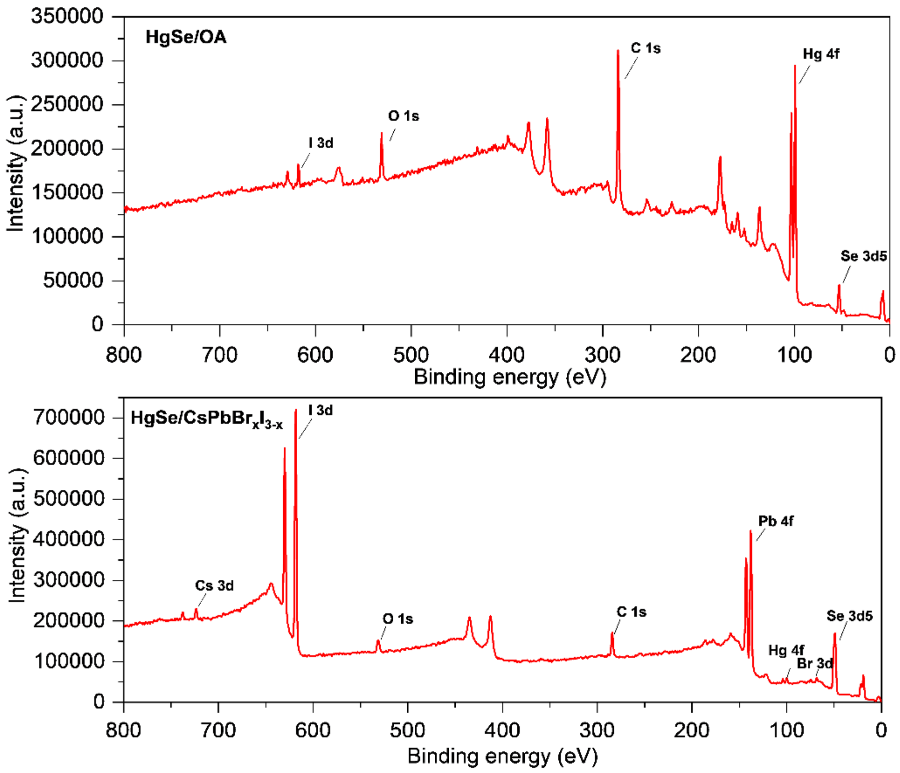

Figure S2. Total XPS spectra of HgSe and HgSe/CsPbBr$_x$I$_{3-x}$

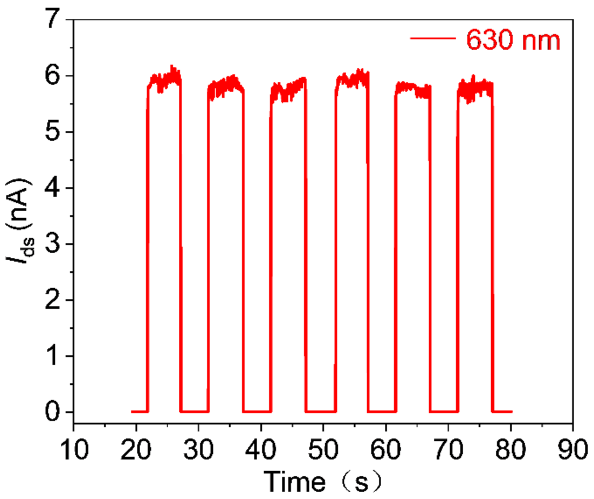

Figure S3. I-T switching curves of CsPbBr$_x$I$_{3-x}$
